# Regulation of triglyceride metabolism by glucocorticoid receptor

**DOI:** 10.1186/2045-3701-2-19

**Published:** 2012-05-28

**Authors:** Jen-Chywan Wang, Nora E Gray, Taiyi Kuo, Charles A Harris

**Affiliations:** 1 Department of Nutritional Science & Toxicology, University of California at Berkeley, Berkeley, CA, 94720, USA; 2 Graduate Program of Metabolic Biology, University of California at Berkeley; 3 Graduate Program of Endocrinology, University of California at Berkeley; 4 Gladstone Institute for Cardiovascular Disease, , San Francisco, CA, 94158, USA; 5 Department of Medicine, University of California, San Francisco, CA, 94143, USA

**Keywords:** Glucocorticoid, Glucocorticoid receptor, Triglyceride, Lipogenesis, Lipolysis, Glucocorticoid response Element, Transcription

## Abstract

Glucocorticoids are steroid hormones that play critical and complex roles in the regulation of triglyceride (TG) homeostasis. Depending on physiological states, glucocorticoids can modulate both TG synthesis and hydrolysis. More intriguingly, glucocorticoids can concurrently affect these two processes in adipocytes. The metabolic effects of glucocorticoids are conferred by intracellular glucocorticoid receptors (GR). GR is a transcription factor that, upon binding to glucocorticoids, regulates the transcriptional rate of specific genes. These GR primary target genes further initiate the physiological and pathological responses of glucocorticoids. In this article, we overview glucocorticoid-regulated genes, especially those potential GR primary target genes, involved in glucocorticoid-regulated TG metabolism. We also discuss transcriptional regulators that could act with GR to participate in these processes. This knowledge is not only important for the fundamental understanding of steroid hormone actions, but also are essential for future therapeutic interventions against metabolic diseases associated with aberrant glucocorticoid signaling, such as insulin resistance, dyslipidemia, central obesity and hepatic steatosis.

## Introduction

The balance of lipogenesis and lipolysis is vital for maintaining triglyceride (TG) homeostasis in mammals. Several endocrine systems play important roles in these processes. Glucocorticoids are steroid hormones that carry critical and complex actions in TG metabolism. During fasting and starvation, increased levels of glucocorticoids in circulation stimulates lipolysis in adipocytes, and TG is hydrolyzed to fatty acids (FA) and glycerol. Free FA (FFA) are mobilized to skeletal muscle and liver to be oxidized and used as energy, whereas glycerol becomes the substrate for hepatic gluconeogenesis. Such metabolic adaptation is critical for the survival of mammals during fasting and starvation. During the fed state, glucocorticoids participate in lipid metabolism for several physiological purposes. Animals starved for a period of 36–56 hr and then fed with high glucose diet showed an increase of hepatic *de novo* lipogenesis (DNL) [1]. Intriguingly, these effects are reduced upon adrenalectomy and restored with glucocorticoid replacement. Thus, glucocorticoids are required for feed efficiency [1-4]. Notably, patients with Cushing’s syndrome, which is characterized by elevated circulating cortisol levels, have fat accumulation in certain depots, such as the central abdomen, the supraclavicular region the face, and the dorsocervical region, the pathognomonic “buffalo’s hump”. However, fat storage is decreased in subcutaneous depots of the extremities [5,6]. These observations highlight that glucocorticoids likely affects both lipogenesis and lipolysis, though depending on different fat depots. Although there is increased fat mass overall, Cushing’s syndrome could be accounted for as a result of fat redistribution. In fact, using stable isotope labeling technique, it is confirmed that white adipose tissues (WAT) of mice treated with dexamethasone (DEX, a synthetic glucocorticoid) for 4 days have an increased TG synthesis and lipolysis concurrently [7]. Why do glucocorticoids concurrently increase TG synthesis and lipolysis in a futile cycle? By increasing both the synthesis and lipolysis of TG, glucocorticoids could have turned up the gain of additional hormonal signals that would inhibit TG synthesis or lipolysis alone.

While glucocorticoids are important for the regulation of lipid homeostasis in different physiological states, excess and/or chronic glucocorticoid exposure can cause lipid disorders, such as central obesity, dyslipidemia and fatty liver [5,8]. Excess/chronic glucocorticoid-induced insulin resistance could partially result from a primary disruption of lipid homeostasis. Furthermore, excess/chronic glucocorticoids increase the circulating FFA and induce ectopic lipid accumulation in skeletal muscle and liver, all are associated with insulin resistance [9,10]. In contrast, suppressing glucocorticoid action in vivo, such as by inhibiting 11β hydroxysteroid dehydrogenase type 1 (11β-HSD 1, which converts inactive glucocorticoids to active ones) or activating 11β-HSD 2 (which converts active glucocorticoids to inactive ones), improves lipid profiles and insulin sensitivity [11-17].

The biological effects of glucocorticoids are mainly conferred by their cognate intracellular receptor, the glucocorticoid receptor (GR). Upon binding to ligands, GR associates with genomic glucocorticoid response elements (GRE) to modulate the transcription of nearby genes (called GR primary target genes), which in turn trigger the biological effects of glucocorticoids. GR can directly bind to the genomic GRE. Alternatively, GR can occupy the GRE indirectly through association with other DNA-binding transcription factors. Such a GRE is called a tethering GRE [18,19]. Recent studies applying chromatin immunoprecipitation sequencing (ChIPseq) have identified genome-wide GR binding regions (GBRs) in distinct cell types [7,20-22]. In these GBRs, the classical GRE sequences are highly represented, albeit small differences exist between them [7,20-22] (Table 1). In any case, the key to understanding the mechanisms of glucocorticoid-regulated TG homeostasis is to identify GR primary target genes modulating lipid metabolism and, furthermore, to elucidate the mechanisms of glucocorticoid-regulated transcription of these primary targets. Here, we overview the current knowledge of glucocorticoid-regulated genes, including their specific role in metabolic function of glucocorticoids and the mechanisms governing their transcriptional regulation in TG metabolism.

**Table 1:** Consensus GRE sequences identified from ChIPseq experiments

Cell Type	Sequences	Reference
A549 human lung epithelial	**RGNACANNNTGTNCY**	[22]
3 T3-L1 mouse Adipocyte	**NNNACWNNNTGTNYY**	[7]
3 T3-L1 mouse predipocyte	**RGNACANNNTGTNCY**	[20]
3 T3-L1 mouse predipocyte	**RGNACWSNVWGTKCY**	[21]

**R: A** or **G, Y**: **C** or **T**, **W: A** or **T, S: G** or **C, K: G** or **T, N:** any nucleotide.

### GR-regulated genes in lipogenic action

#### Acetyl-CoA carboxylase and fatty acid synthase

**Figure 1:**
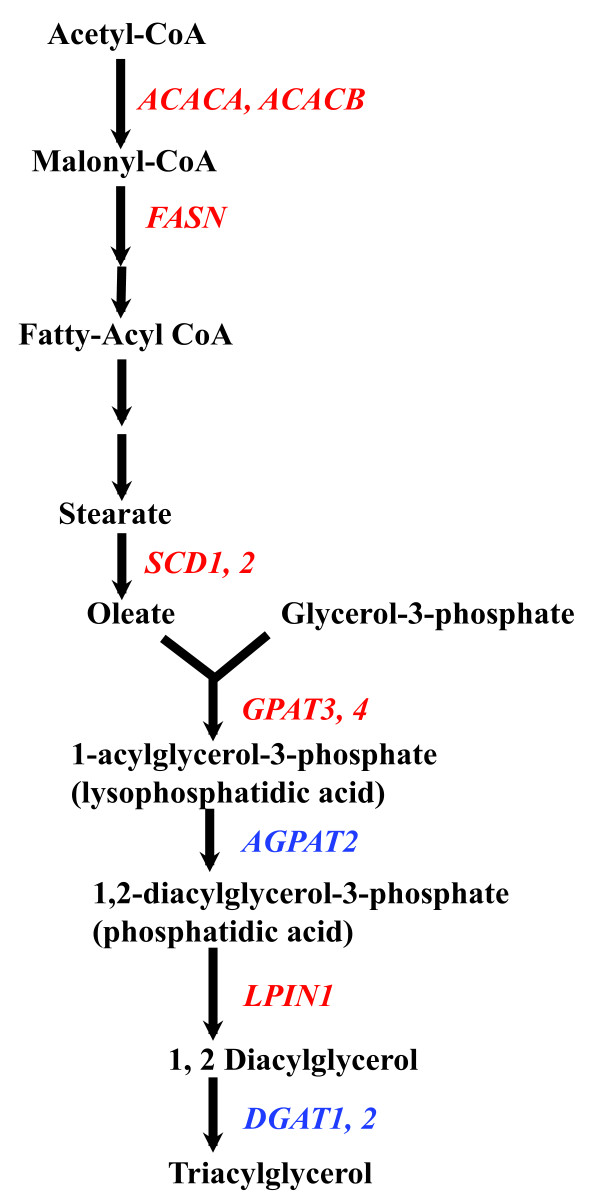
**Glucocorticoid-regulated genes in lipogenic pathway.** Red indicates potential GR primary target genes, whereas blue indicates genes regulated by glucocortiocids with no GBR is identified in or nearby their genomic regions.

Glucocorticoids regulate several genes encoding enzymes in DNL and TG synthesis (Figure 1). *Acetyl-CoA Carboxylase 1* and *2* (*ACACA* and *ACACB*) encode rate-controlling enzymes in the FA synthesis pathway. Glucocorticoids have been shown to increase the expression of *ACACA* and *ACACB in vitro* and *in vivo*[23-25]. The human *ACACA* and *ACACB* genes contain 3 (PI-III) and 2 (PI-II) alternative promoters, respectively [26]. A recent report showed that the human *ACACA* and *ACACB* gene promoters can be activated by glucocorticoids when they are inserted into a synthetic reporter gene [26]. The exact location of the GRE in human *ACACA* and *ACACB* gene were not reported. With chromatin immunoprecipitation sequencing (ChIPseq), GR binding regions (GBR) in or nearby the mouse *Acaca* and *Acacb* genes in 3 T3-L1 mouse adipocytes were identified [7]. For the mouse *Acaca* gene, a single GBR is found in the intron [7]. For the mouse *Acacb* gene, one GBR is identified upstream of the transcription start site (TSS), whereas multiple GBR are found in intronic regions [7]. The ability of these GBR to mediate glucocorticoid-induced changes in *Acaca* and *Acacb* gene expression has not yet been examined.

*Fatty acid synthase* (*FASN*) encodes another rate-controlling enzyme in lipogenesis. The regulation of *FASN* by glucocorticoids has been reported in many tissues, including liver, adipose tissues, and lung [27]. A previous study showed that glucocorticoid treatment increases the activity of a reporter gene that contains −1592 to +65 (relative to TSS) of human *FASN* gene [28]. Thus, the GRE(s) that mediate the stimulatory effect of glucocorticoids on *FASN* gene lies within this −1592 to +65 region. Transgenic mice harboring a reporter gene that contains 2.1 kb rat *Fasn* gene promoter fused to chloramphenicol acetyltransferase (CAT) were generated to study the regulation of the *Fasn* gene *in vivo*. DEX treatment increases the reporter gene in multiple tissues, including liver, white adiopose tissue (WAT), brown adipose tissue (BAT), and lung [29]. This finding suggests that the GRE(s) lies within this 2.1-kb region upstream from TSS in the rat *Fasn* gene. For the mouse *Fasn* gene, ChIPseq identified an intronic GBR located within +3248 to +3463 (relative to TSS) region [7]. The function of this mouse *Fasn* GBR has not yet been studied.

A recent report shows that despite the observation of the induction of *ACACA, ACACB* and *FASN* gene expression by corticosterone in a human Chub-S7 adipocyte cell line, lipogenesis is not increased, but decreased [24]. It is likely due to increased phosphorylation of ACACA on serine 79/218 which reduces its activity [24]. However, when insulin is present in cultured medium, corticosterone enhances insulin-stimulated lipogenesis in these cells [24]. The positive cross-talk between glucocorticoids and insulin in the regulation of lipogenesis has long been reported, and some studies suggest that glucocorticoids’ effect on DNL is dependent on insulin [30,31]. In primary hepatocytes, glucocorticoids have a small or no effect on FA synthesis. They either play a permissive role for insulin to induce FA synthesis or enhance the insulin effect on FA synthesis [30-32]. In agreement with these observations, glucocorticoids and insulin have been shown to have additive or synergistic effects on *ACACA* and *ACACB* gene expression [26,33]. These two hormones also have additive effects on reporter genes that harbor *ACACA* PI and *ACACB* PI promoter (approximately 2 kb for each promoter) [26]. A similar positive cross-talk between glucocorticoids and insulin is found on *FASN* gene regulation [27,32], as they additively activate a reporter gene harboring human *FASN* gene promoter [32].

#### Genes encoding enzymes in TG synthesis

Glucocorticoids activate the expression of several genes encoding enzymes in TG synthesis. GR ChIPseq experiments in 3 T3-L1 adipocytes identified GBR in *Scd1, Scd2, Gpat3, Gpat4, Agpat2* and *Lpin1* (Figure 1)[7]. The expression of these genes is also increased by DEX treatment in 3 T3-L1 adipocytes and mice WAT [7]. When these GBR are inserted into a reporter plasmid, GBR of all but *Agpat2* can mediate glucocorticoid response [7]. These data suggest that all of them are likely GR primary target genes, though the GRE in these GBR have not been identified. Additional genes encoding enzymes in TG synthetic pathways, such as *Dgat1* and *Dgat2* (Figure 1), have been shown to be regulated by glucocorticoids [15,34]. However, it is unclear whether they are GR primary target genes, as no GBR near their genomic regions is identified so far. Furthermore, a study identified a GRE located between −311 and −297 of mouse *Lpin1* gene [35]. The location of this GRE is different from the 4 GBR identified from our ChIPseq experiment, which are located between −1099 and −1320, -1709 and −1937, −27515 and −27845, and −28575 and −29617 [7]. The latter two GBR conferred glucocorticoid response in reporter assays. It is likely that multiple GREs are involved in glucocorticoid-activated *Lpin1* gene transcription.

Notably, the precise purpose of glucocorticoids to induce the transcription of these TG synthesis genes has not yet been well established. Among these glucocorticoid-regulated genes discussed above, the positive effects of glucocorticoids on the activity of Lpin1, which contains phosphatidate phosphatase (PAP1) activity, [36,37] and Scd1 and Scd2, which possess stearoyll-CoA desaturase activity, have been reported [38]. The effects of glucocorticoids on the activity of Gpat3, Gpat4 and Agpat2 are unknown. Dolinsky et al. found that DGAT1 and DGAT2 expression in the liver was increased about 60% and DGAT activity was increased approximately 20% by DEX [34]. Similar to their effect on FA synthesis, glucocorticoids act with insulin to stimulate TG synthesis. The increase of TG synthesis by glucocorticoids alone in hepatocytes was reported in some studies [39] but not all [40]. However, the additive or synergistic effect of glucocorticoids and insulin on TG synthesis is observed in most reports [39,40]. Glucocorticoids and insulin both increase the transcription of *Scd1* gene [41]. Furthermore, insulin induces the phosphorylation of Gpat3 and Gpat4, which enhances their enzymatic activities [42]. Glucocorticoids and insulin also act together to increase the expression of lipoprotein lipase (*LPL*), which hydrolyzes extracellular TG to FFA. These FFA can be taken up by adipoyctes and enter the lipogenic pathway [43,44]. It has been suggested that insulin increases the stability of *Lpl* mRNA and the activity of Lpl in mouse 3 T3-L1 adipocytes and human primary pre-adipocytes [45]. Glucocorticoids increase the *Lpl* mRNA levels in 3 T3-L1 adipocytes and human primary pre-adipocytes [45], but the mechanisms underlying these effects are unknown. ChIPseq experiments identified a GBR located 14 kb upstream of *Lpl* TSS in 3 T3-L1 adipocytes [7]. The ability of this GBR to mediate glucocorticoid responses has not been investigated. The only gene in the lipogenic pathway whose expression is inhibited by insulin is *Lpin 1*[46]. The metabolic significance of this effect is not entirely clear. *Lpin1* expression is induced upon fasting, and Lpin1 protein has been shown to participate in transcriptional regulation of genes involved in FA oxidation, a critical step in metabolic adaptation during fasting [47,48]. This function of Lpin1 appears to be independent of its PAP1 activity.

### GR-regulated genes in lipolytic action

#### Genes encoding enzymes in adipocyte lipolysis

**Figure 2:**
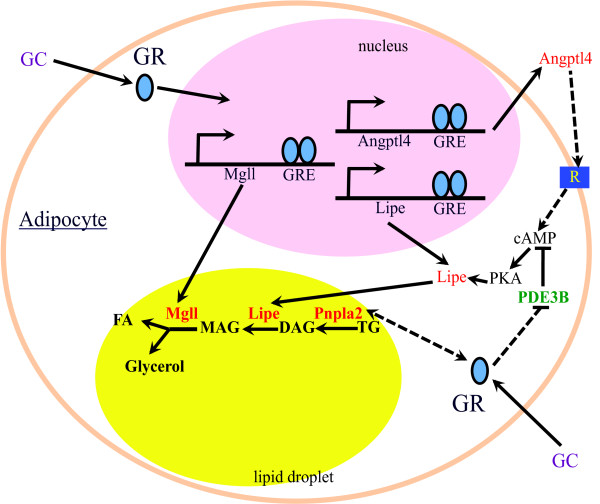
**Glucocorticoids promote lipolysis in adipocytes.** Glucocorticoids (GC) activate the transcription of *Lipe, Mgll,* and *Angptl4* gene transcription. *Lipe* and *Mgll* encode enzymes in the lipolytic pathway. Angptl4 is a secreted protein that likely binds to an unknown receptor to increase cAMP levels in adipocytes, which in turn activates PKA. PKA phosphorylates Lipe, which increases its activity and translocation from cytosol to lipid droplet. GC also increase the expression of Pnpla2 and decrease the expression of Pde3b. The mechanisms for these events are unknown (dashed line).

Glucocorticoids have been shown to elevate the expression of genes encoding all three enzymes in the lipolytic pathway in adipocytes [7,49]. *PNPLA2* (also called *ATGL* or *desnutrin*) encodes an enzyme that hydrolyzes TG to diacyglycerol (DAG) (Figure 2). *LIPE* (a.k.a. hormone-sensitive lipase) encodes an enzyme that hydrolyzes DAG to monoacylglycerol (MAG), whereas *MGLL* (monoacylglycerol lipase) encodes an enzyme that hydrolyzes MAG to glycerol (Figure 2). The GBR of mouse *Lipe* and *Mgll* genes are identified by ChIPseq, and these GBR can mediate glucocorticoid response when inserted into a synthetic reporter plasmid [7]. Thus, *Lipe* and *Mgll* are likely GR primary target genes. The GBR of *PNPLA2* gene has not been identified. Notably, mouse *Pnpla2* gene is positively regulated by a member of FoxO transcription factors, FoxO1, in 3T3-L1 adipocytes [50]. Glucocorticoids have been shown to increase the expression of FoxO1 and/or FoxO3, another member of the FoxO family, in WAT and other tissues [51-53]. Therefore, glucocorticoids might activate *Pnpla2* gene transcription indirectly through the elevation of FoxO1 and FoxO3. However, this model requires confirmation.

It is important to note that insulin represses the expression and/or the activity of proteins involved in lipolysis, such as *Pnpla2*[54,55] and *Lipe*[56,57]. This observation of insulin effect is in contrast to genes involved in lipogenesis discussed above.

#### Phosphodiesterase 3B, cGMP-inhibited (PDE3B)

In addition to lipolytic enzymes, the cAMP signaling pathway plays a key role in the induction of adipocyte lipolysis. cAMP activates protein kinase A (PKA) that phosphorylates LIPE. This phosphorylation increases LIPE enzymatic activity and translocates LIPE from cytosol to lipid droplet (Figure 2) [58]. PKA also phosphorylates PLIN1, which can lead to the stimulation of lipolysis. The details of this pathway are discussed in several recent reviews [57,59,60]. Glucocorticoid treatment has been shown to increase cAMP levels in several cell types. However, unlike in neurons where glucocorticoids rapidly increase intracellular cAMP levels through a non-genomic mechanism [61], in adipocytes the induction of cAMP levels requires several hours of glucocorticoid treatment [25,62]. Thus, the glucocorticoid-elevated cAMP in adipocytes likely requires the induction of gene transcription and subsequent protein synthesis. At least two mechanisms are proposed. One is the inhibition of the expression of *PDE3B* (Figure 2) [25]; the other is the induction of *angiopoietin-like 4* (*ANGPTL4*, a.k.a. *fasting-induced adipose factor, FIAF*) (Figure 2), which will be discussed later in this review.

*PDE3B* encodes a protein that decreases cAMP signaling pathway by hydrolyzing cAMP to AMP in adipocytes. Insulin inhibits PDE3B activity through both phosphoinositide 3-kinase (PI3K)/Akt dependent and independent [63,64] pathways to suppress lipolysis. The suppression of the *Pde3b* expression by glucocorticoids is found both in vivo and in vitro [25,62], and this suppressive effect could contribute to increased cAMP levels in adipocytes and the subsequent induction of lipolysis. However, how glucocorticoids repress *Pde3b* gene expression has not been studied.

#### Carboxyesterase 1d (Ces1d, a.k.a. Triacylglycerol hydrrolase, TGH)

Dolinsky et al. examined the effects of DEX on hepatic TG metabolism [34]. Specifically, they focused on the process where TG, stored in intracellular droplets, are lipolyzed prior to re-esterification into a VLDL particle. They observed no effect of DEX on hepatic VLDL secretion *in vivo* or *in vitro*, but did find a 50% decrease in TG turnover in hepatocytes with DEX treatment. Ces1d is an enzyme believed to mediate the lipolysis of TG in the liver. *Ces1d* mRNA levels were decreased by DEX treatment, but this repression was not due to an effect on transactivation, as no difference was seen in nuclear run-on experiments and the *Ces1d* promoter did not confer DEX responsiveness in reporter assays. Instead, it was found that DEX reduced *Ces1d* mRNA stability via a 3′ UTR element. These experiments further demonstrate the complexity of glucocorticoid action on TG metabolism.

#### Angptl4

Recent studies also identify *Angptl4* as a GR primary target gene participating in lipid metabolism in liver and WAT [65]. The *Angptl4* gene encodes a secreted protein that inhibits extraceullular LPL and promotes intracellular lipolysis in adipocytes [62,66,67]. The combination of these two effects results in the mobilization of lipids from adipocytes to plasma. *Angptl4* null mice (*Angptl4*^*−/−*^) are protected from excess glucocorticoid-induced hepatic steatosis and hyperlipidemia [65]. These lipid disorders are attributed, at least in part, to the ability of glucocorticoids to redistribute lipids from adipocytes to hepatocytes. Indeed, ANGPTL4 has been shown to participate in glucocorticoid-induced adipocyte lipolysis (Figure 2) [62], as DEX-stimulated lipolysis is impaired in WAT of *Angptl4*^*−/−*^ mice [62]. Intriguingly, purified human ANGPTL4 protein (hANGPTL4) can directly increase adipocyte lipolysis [62].

Circulating glucocorticoid levels are elevated during fasting. Treatment with RU486, an antagonist of GR, decreases 24 hr fasting-induced WAT lipolysis. It highlights the important role of glucocorticoids in fasting-induced WAT lipolysis [62]. *Angptl4* gene expression is highly induced upon fasting, an effect that is attenuated by RU486 treatment [62]. Therefore, glucocorticoids play a key role in the elevation of *Angptl4* gene expression during fasting. The fact that WAT from *Angptl4*^−/−^ mice has reduced lipolysis with 24 hr fasting [62] further demonstrates the importance of the glucocorticoid-Angptl4 axis in fasting-induced lipolysis in WAT. In summary, during fasting, glucocorticoids activate *Angptl4* gene expression, which in turn promotes lipolysis in adipocytes.

Since Angptl4 is a secreted protein, how does it affect adipocyte lipolysis? Purified hANGPTL4 increases cAMP levels in adipocytes (Figure 2) [62]. Both fasting and DEX treatment for 24 hr increases cAMP levels in WAT, but this induction is impaired in *Angptl4*^*−/−*^ WAT [62]. Thus, ANGPTL4 promotes lipolysis through activating cAMP pathway. The receptor of ANGPTL4 in adipocytes; however, has not been identified. Therefore, the intracellular signaling events that lead to the increase of cAMP production are unclear. To date, ANGPTL4 has been shown to interact with fibronectin, vitronectin and integrin β1β5 in keratinocytes to activate integrin-mediated signaling [68,69]. Angptl4 also affects other signaling molecules, such as RAS/ERK [70] and AMP-activated protein kinase (AMPK) [71] in endothelial cells and hippocampus, respectively. However, whether Angptl4 employs such mechanisms in adipocytes has not been examined.

The GRE of rat *Angptl4* gene is located in the 3′ untranslated region, between +6227 and +6441 [65]. DEX treatment increases DNase I sensitivity and histone H4 hyperacetylation in this region. Notably, this GRE is highly conserved between rat, mouse and human [65]. In the human *Angptl4* gene, another GBR is identified approximately 8 kb upstream from the TSS [72]. Interestingly, insulin has been shown to decrease the expression of *Angptl4* gene [73]. This response is consistent with the metabolic effect of insulin, which represses adipocyte lipolysis, and the fact that insulin resistance is highly associated with hyperlipidemia [74,75].

#### Hes1

Glucocorticoids repress the transcription of a transcriptional repressor, *Hes1*[76]. Overexpression of Hes1 increases the expression of *pancreatic lipase* (*Pnlip*) and *pancreatic lipase-related protein* (*Pnliprp2*). In liver, Pnlip and Pnliprp2 contribute to TG hydrolysis and the subsequent stimulation of fatty acid oxidation and ketogenesis [77]. Therefore, glucocorticoid-mediated inhibition of the transcription of *Hes1* results in a decreased expression of *Pnlip* and *Pnliprp2*, which in turn elevates hepatic TG accumulation [76]. It is unclear whether Hes1 directly regulates the transcription of *Pnlip* and *Pnliprp2,* as Hes1 usually serves as a transcriptional repressor. In contrast, *Hes1* appears to be a GR primary target gene. The GRE of the mouse *Hes1* gene is located between −463 and −414 (relative to TSS) [76]. A reporter gene that contains −463 to +46 region of the *Hes1* genomic region is suppressed by DEX, whereas a reporter that contains −414 to +46 region does not respond to DEX [76]. In addition, a consensus GRE half-site (TGTTCC) was found between −463 and −414, and GR likely exerts this repressive effect through direct DNA binding to this half-site. In cells overexpressing wild type GR, DEX treatment reduces the activity of the reporter containing −463 to +46 of *Hes1* gene [76]. In cells overexpressing GR mutants with defective DNA binding ability, DEX can no longer suppress the reporter gene activity [76]. ChIP experiments further confirm the recruitment of GR to this −463 to +46 region upon DEX treatment. HDAC1, a transcriptional corepressor, is also recruited to this region upon DEX treatment [76]. Presumably, HDAC1 interacts with GR and is responsible for the inhibition of *Hes1* gene transcription. Thus, *Hes1* is likely a GR primary target gene that controls hepatic TG storage by regulating genes involved in TG hydrolysis in the liver.

### GR-regulated genes involved in bile acid metabolism

#### Na+−taurocholate transport protein (Ntcp/Slc10A1)

Bile acids (BA) are important to dietary fat digestion. They also serve as ligands for farnesoid X receptor (Fxr), which regulates TG metabolism. The role of Fxr in TG metabolism is discussed in a recent review [78]. In liver-specific GR knock-down mice, BA content in gallbladder is decreased [79]. Liver BA uptake is impaired and intestinal fat absorption was defective in these mice as there was greater fecal loss of TG and FFA. Furthermore, these mice promote greater thermogenic potential as shown by increased cAMP signaling in BAT and higher thermogenic gene expression [79]. These phenotypes are likely attributed, at least in part, to a lower expression of the major hepatocyte basolateral BA transporter, *Nctp*[79]. Nctp affects dietary fat absorption and brown fat activation. Although the GRE of *Nctp* has not been identified, ChIP shows GR occupancy at the proximal promoter of *Nctp* gene [79]. In mice harboring a GR mutant that has a mutated dimerization domain and altered DNA binding ability (GR^dim^), glucocorticoids do not activate *Ntcp* gene expression [79]. These results suggest that the ability of GR to bind to the GRE is critical for the induction of *Ntcp* by glucocorticoids.

### Transcriptional regulators that participate in glucocorticoid-regulated lipid metabolism

GR regulates the transcription of specific genes by interacting with a wide variety of transcription regulators; however, only few have been shown to participate in glucocorticoid-regulated transcription of genes involved in TG metabolism. Here we discuss two transcriptional regulators that play a role in glucocorticoid-regulated TG metabolism.

#### Med1

Med1, a component of Mediator cofactor complex, has been shown to associate and coactivate with several nuclear receptors, including GR [80-82]. Gene expression analysis in Med1 knockout mouse embryonic fibroblast cells shows that Med1 is selectively required for the regulation of GR target genes. Med1 contains two LXXLL motifs that are involved in the interaction with GR [80]. Overexpression of Med1 enhances glucocorticoid-activated transcription from an MMTV promoter-based reporter gene. Mutations of these LXXLL motifs attenuate but do not eliminate this enhancement [80]. Indeed, it has been shown that Med1 could be recruited by GR through another pathway. The N-terminus of Med1 interacts with a transcriptional coactivator, CCAR1, which in turn associates with other transcription coactivators, CoCoA-NCoA1. NCoA1 can directly associate with the ligand binding domain of GR. The fact that depletion of CCAR1 in cells reduces ligand-dependent recruitment of Med1 to the GR-target gene promoters confirm the importance of this mechanism for GR-Med1 interaction [83].

Excess DEX-induced hepatic steatosis is reduced in liver-specific *Med1* knockout mice (*Med1*^*Δliver*^). In WT mice, DEX represses the expression of *medium-* and *short-chain acyl-CoA dehydrogenases (Mcad* and *Scad*), which encodes enzymes involved in FA oxidation. In *Med1*^*Δliver*^ mice, the repression of *Mcad* and *Scad* by DEX treatment is reduced [84]. Therefore, an increased expression of genes involved in FA oxidation reduces TG accumulation induced by DEX in the livers of *Med1*^*Δliver*^ mice. Whether *Mcad* and/or *Scad* are GR primary target genes are unclear. Med1 usually serves as a transcriptional coactivator. However, certain coactivators, such as GRIP1/TIF-2, can also act as a corepressor [85].

Using small hairpin RNA (shRNA) to reduce the expression of GR in liver, the expressions of genes involved in FA oxidation, such as *carnitine palmitoyltransferase 1*α *(Cpt1*α*)* and *acetyl-CoA acyltransferase 2 (Acaa2)*, are increased [76]. These results suggest that GR negatively regulates hepatic FA oxidation gene expressions. Considering that the disruption of hepatic Med1 also causes elevation of genes involved in FA oxidation, it suggests a link between GR and Med1 in the aspect of regulating genes in FA oxidation. To test this model, one should verify whether any of these GR- or Med1-regulated genes involved in FA oxidation are GR primary target genes, and then whether Med1 is recruited to their GRE.

#### Liver X receptor (LXR)

A recent report shows that glucocorticoid-induced hepatic steatosis is impaired in liver X receptor β null mice (Lxrβ^−/−^) [86]. Lxrβ is not a glucocorticoid-regulated gene. However, Lxrβ is required for glucocorticoid-induced GR recruitment to the GRE of phosphoenolpyruvate carboxykinase (*Pepck*) gene [86], which encodes a rate-controlling enzyme in gluconeogenesis. Thus, Lxrβ seems to act as a coactivator for GR to activate gluconeogenic genes. How Lxrβ exerts this effect is unclear. Interestingly, Lxrβ appears to affect glucocorticoid-regulated lipid metabolism by modulating glucose metabolism and insulin sensitivity. Compared to glucocorticoid-treated WT mice, glucocorticoid-treated Lxrβ^−/−^ mice are more insulin sensitive [86]. This observation could be an explanation for decreased glucocorticoid-induced TG accumulation in Lxrβ^−/−^ mice.

Interestingly, another recent report showed that treating hepatoma cells with LXR ligands suppresses glucocorticoid-induced *Pepck *and *glucose-6-phosphatase* (*G6Pase)* gene expression [87]. Microarray analyses showed that LXR ligand only affects a subset of glucocorticoid-regulated genes. Both gel shift and ChIP experiments suggest that LXRα/RXRα heterodimer competes with GR to bind to the GRE of rat *G6pase* gene [87]. In agreement with its effects on DEX-induced gluconeogenic gene expression, treating rats with a LXR ligand attenuates DEX-increased plasma glucose levels [87]. Thus, endogenous unliganded LXRβ is required for a maximal GR occupancy on the GRE of gluconeogic genes (as discussed in previous paragraph), whereas LXR ligands appear to suppress glucocorticoid-activated gluconeogic gene transcription by inhibiting the recruitment of GR to the GREs of these genes.

## Conclusion

In this manuscript, we review glucocorticoid-regulated genes participating in the modulation of TG metabolism. In the last decade, with the development of gene expression arrays and ChIPseq methodologies, the number of identified potential GR primary target genes has significantly increased. However, it is important to note that only a handful of identified GR primary target genes have been shown to mediate glucocorticoid effects on TG metabolism *in vivo*. The on-going identification of such “causative primary response genes” (CPRG) is critical for further elucidation of mechanisms governing the metabolic effects of glucocorticoids.

Although GBR are being identified in certain glucocorticoid-regulated genes involved in TG metabolism, more studies are required to verify the role of these GBR in conferring glucocorticoid responses. It could pose a challenge, as most GBR identified from ChIPseq are far away from TSS, whereas only less than 10% of GBR are located within 5 kb upstream of TSS [7,22,72]. The traditional approach focused on this promoter region to identify the GRE of glucocorticoid-regulated genes. Although inserting GBR into a synthetic reporter gene to study their enhancer activity is commonly used, the context of such reporter plasmids could be very different from the endogenous genomic structure. More efforts are required to learn the exact role of long-range GBR/GRE in the regulation of specific glucocorticoid-regulated genes.

It is well established that most chromosomal GRE are composite elements, in which non-GR transcriptional regulators act with GR to confer a complete hormonal response [88,89]. Therefore, transcriptional regulatory complexes, which include GR, non-GR DNA binding regulators and non-DNA binding cofactors, assembled on each GRE are likely to be distinct. This suggests that certain cofactors may be required only for the transcriptional regulation of a subset of glucocorticoid target genes. Thus, uncovering the specific molecular features of each GR-containing regulatory complex shall provide novel and specific targets for therapeutic development. The dissection of transcriptional mechanisms could also elucidate the molecular basis of the cross-talk between glucocorticoids and insulin, specifically, their synergistic effects on the expression of lipogenic genes and their antagonistic effects on the expression of lipolytic genes. It is possible that the cross-talk between these two hormones are specified by the interaction between GR and specific transcription factors. For example, FoxO1 and FoxO3 have been shown to play an accessory role in assisting glucocorticoid response and mediating the repressive effect of insulin in certain genes, such as *Pepck* and *G6Pase*[90,91]. Thus, glucocorticoid-activated genes containing FoxO binding site nearby the GRE may be usually suppressed by insulin. Transcription factors that participate in insulin action and functionally interact with GR to regulate TG metabolism genes are difficult to speculate, as most of their GRE(s) have not been identified. Future studies should address these important issues.

## Competing interests

None of the authors had any financial and personal relationships with other people or organizations that could inappropriately influence their work.

## Authors’ contributions

JCW, NEG, TK and CH wrote the manuscript. All authors read and approve the manuscript.
